# Divergence with gene flow across a speciation continuum of *Heliconius* butterflies

**DOI:** 10.1186/s12862-015-0486-y

**Published:** 2015-09-24

**Authors:** Megan A. Supple, Riccardo Papa, Heather M. Hines, W. Owen McMillan, Brian A. Counterman

**Affiliations:** Smithsonian Tropical Research Institute, Panamá City, Panamá; Biomathematics Program, North Carolina State University, Raleigh, NC 27695 USA; Research School of Biology, The Australian National University, 134 Linnaeus Way, Canberra, ACT 2601 Australia; Department of Biology and Center for Applied Tropical Ecology and Conservation, University of Puerto Rico—Rio Piedras, 00931 San Juan, Puerto Rico; Department of Biology, Pennsylvania State University, 208 Mueller Laboratory, University Park, PA 16802 USA; Department of Biological Sciences, Mississippi State University, 295 Lee Boulevard, Mississippi State, MS 39762 USA

**Keywords:** Speciation genomics, Speciation continuum, Genomic divergence, *Heliconius*

## Abstract

**Background:**

A key to understanding the origins of species is determining the evolutionary processes that drive the patterns of genomic divergence during speciation. New genomic technologies enable the study of high-resolution genomic patterns of divergence across natural speciation continua, where taxa pairs with different levels of reproductive isolation can be used as proxies for different stages of speciation. Empirical studies of these speciation continua can provide valuable insights into how genomes diverge during speciation.

**Methods:**

We examine variation across a handful of genomic regions in parapatric and allopatric populations of *Heliconius* butterflies with varying levels of reproductive isolation. Genome sequences were mapped to 2.2-Mb of the *H. erato* genome, including 1-Mb across the red color pattern locus and multiple regions unlinked to color pattern variation.

**Results:**

Phylogenetic analyses reveal a speciation continuum of pairs of hybridizing races and incipient species in the *Heliconius erato* clade. Comparisons of hybridizing pairs of divergently colored races and incipient species reveal that genomic divergence increases with ecological and reproductive isolation, not only across the locus responsible for adaptive variation in red wing coloration, but also at genomic regions unlinked to color pattern.

**Discussion:**

We observe high levels of divergence between the incipient species *H. erato* and *H. himera*, suggesting that divergence may accumulate early in the speciation process. Comparisons of genomic divergence between the incipient species and allopatric races suggest that limited gene flow cannot account for the observed high levels of divergence between the incipient species.

**Conclusions:**

Our results provide a reconstruction of the speciation continuum across the *H. erato* clade and provide insights into the processes that drive genomic divergence during speciation, establishing the H. erato clade as a powerful framework for the study of speciation.

**Electronic supplementary material:**

The online version of this article (doi:10.1186/s12862-015-0486-y) contains supplementary material, which is available to authorized users.

## Background

The ability to study high-resolution genomic patterns of divergence in natural populations has enabled valuable insights supporting a fundamental shift in our understanding of the origins of species. Historically, speciation research considered geographic isolation (allopatry) as the main barrier that allowed populations to diverge and reproductive incompatibilities to evolve. However, more recent theoretical and empirical studies have demonstrated that natural selection can be an important driver of divergence and reproductive isolation, even when populations overlap geographically (parapatry or sympatry) (see [1] for a review). As a result, there has been a shift towards understanding how populations diverge as reproductive isolation increases, but gene flow continues to occur [2]. Genome scans between incompletely reproductively isolated populations and species offer snapshots of the genomic changes that accumulate during the speciation process. Valuable insights into how genomes diverge during speciation come from studies across speciation continua, where taxa pairs with different levels of reproductive isolation can be used as proxies for different stages of speciation.

Initially, metaphorical models invoking selection and differential gene flow were proposed to explain the heterogeneous patterns of genomic divergence that occur as speciation progresses [3]. Two types of genetic hitchhiking, divergence hitchhiking and genomic hitchhiking, were introduced to explain how selection could affect levels of gene flow locally and globally across the genome [4]. Divergence hitchhiking is the result of selection acting on loci responsible for ecological divergence. This causes the local reduction of gene flow in physically linked regions, which leads to the further accumulation of allelic differences around the targets of selection. In genomic hitchhiking, the reduction of gene flow due to divergent selection reduces genome-wide rates of gene flow. This results in the global accumulation of genetic differences. While these two processes are similar and not mutually exclusive, they reflect two fundamentally different ways that genomes may evolve during speciation—through the local accumulation of divergence around targets of selection or through the genome-wide accumulation of divergence [4].

*Heliconius* butterflies offer an exceptional opportunity to explore patterns of divergence at different stages across the speciation continuum. The radiation of warning colors across the genus has resulted in an amazing diversity of wing color patterns within and between species. For example, *Heliconius erato* has radiated into over 25 geographically distinct color pattern races distributed throughout the Americas [5]. When these divergently colored races come into contact, they often hybridize. While races of *H. melpomene* have been shown to mate assortatively based on wing color patterns [6], this has not been demonstrated between races of *H. erato. Heliconius erato* practices pupal mating, which involves males mating with partially eclosed females, thereby reducing the opportunity for wing color based mate choice [7]. Empirical support for the lack of color based assortative mating comes from hybrid zone analysis that shows Hardy-Weinberg equilibrium of wing color patterns [8]. Hybrid zones are characterized by the breakdown of parental color patterns, including the presence of individuals with intermediate color patterns created through multiple generations of backcrossing [8]. The high frequency of these hybrid phenotypes suggests that there is ongoing gene flow between *H. erato* races with overlapping geographic distributions. The taxonomic boundaries between hybridizing *H. erato* races are primarily maintained by strong frequency dependent selection on wing color patterns [9–13]. This selection is driven by Müllerian mimicry, as similarly unpalatable butterflies are more likely to be avoided if they exhibit a more common wing color pattern, which is more readily recognized by predators.

Besides natural selection due to differential predation based on wing color patterns, additional forms of reproductive isolation have been shown to maintain the species boundaries within *Heliconius*. For example, there is strong color pattern based mate preference between the closely related *H. himera* and *H. erato cyrbia* [14–18]. These species are associated with very different habitats that likely also contribute to ecological isolation: *H. himera* is found in very dry habitats at altitudes above 1000 m; whereas, *H. e. cyrbia* is found in wetter forests at elevations below 1200 m [18]. Despite the strong pre-mating isolation and habitat differences between these two species, hybrids comprise 5-10 % of the population in the narrow zones of overlap [16–18]. Crosses between *H. himera* and several *H. erato* color pattern races have shown that F1 and backcrosses are reproductively viable, suggesting that intrinsic postzygotic barriers have not evolved between the species [14].

Previous studies of the evolutionary relationship between *H. erato* and *H. himera* have been equivocal. Early phylogenetic analysis using mtDNA placed the split between *H. himera* and *H. erato* prior to the *H. erato* radiation [19]. Additionally, a study of allozyme divergence between populations of *H. himera* and *H. e. cyrbia* found clear differences in allele frequencies, supporting the hypothesis that *H. himera* and *H. erato* are distinct species [20]. However, an analysis of rapidly evolving nuclear introns placed *H. himera* within the broader *H. erato* radiation, although with low support [21]. That study suggests that *H. himera* is a group of individuals that diverged during the *H. erato* radiation and over time became reproductively isolated from other *H. erato* populations.

**Fig. 1:**
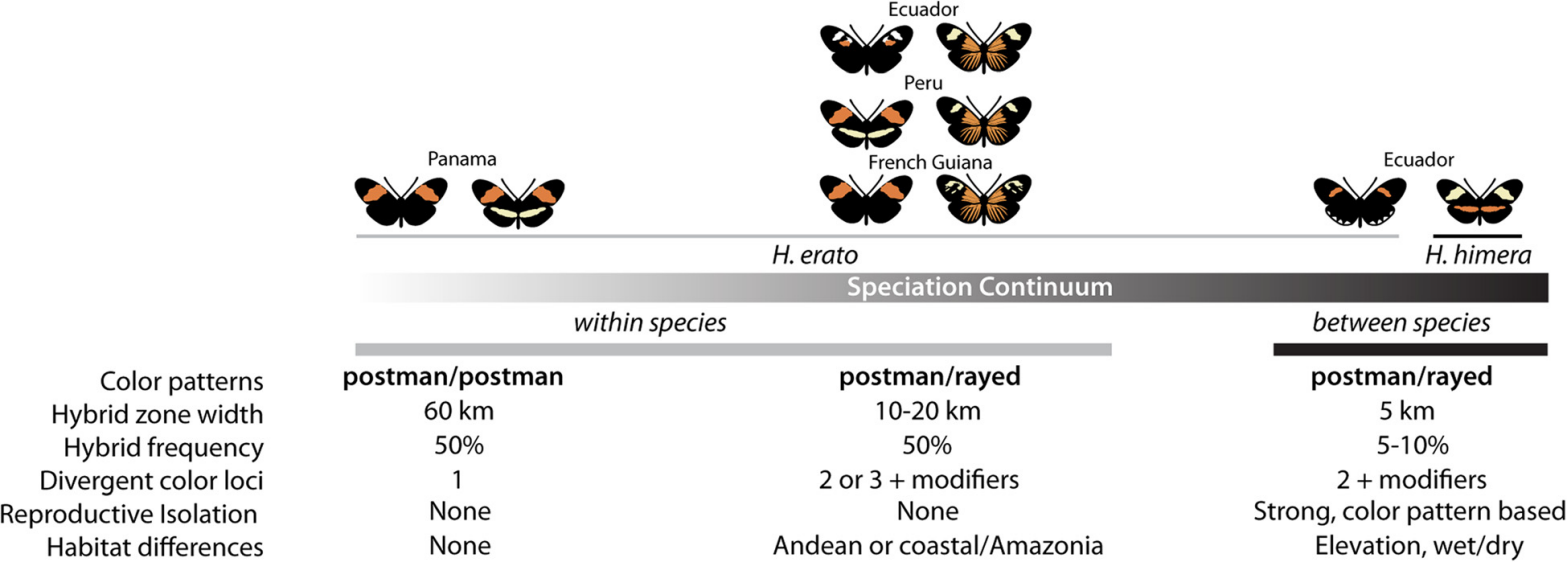
The speciation continuum of the *Heliconius erato* clade. Three stages along the speciation continuum are shown, from a hybridizing pair with no reproductive isolation and minimal color pattern divergence, to a hybridizing pair with strong reproductive isolation and color pattern divergence. For each stage we show details, including color pattern differences [8, 25, 29, 30], the hybrid zone estimates [10, 12, 13, 16–18], the type of reproductive isolation [8, 10, 14–18, 33], and habitat differences [18, 51]. Hybrid zones shown are Panama (*H. e. hydara, H. e. petiverana*), Ecuador (*H. e. notabilis, H. e. lativitta*), Peru (*H. e. favorinus, H. e. emma*), French Guiana (*H. e. hydara, H. e. erato*), Ecuador (*H. e. cyrbia, H. himera*)

There has been substantial progress resolving the genetic basis and evolution of the warning color radiation in *Heliconius* [22–28]. The color pattern variation across the genus is controlled by a handful of unlinked loci that control major differences in wing color patterns. In *H. erato* the adaptive radiation is largely controlled by allelic changes at three unlinked major color pattern loci [29, 30]. The locus controlling red patterning (the *D* locus) is the best characterized and controls the largest changes in warning color. Variation at this locus results in two major color pattern phenotypes: the postman phenotype, which has a red forewing band, and the rayed phenotype, which has a yellow forewing band, red proximal forewing patch, and red hindwing rays (Fig. 1). A combination of linkage analysis, gene expression, and genotype by phenotype association strongly implicate cis-regulatory changes in the transcription factor *optix* as causing variation in red wing pattern elements in *Heliconius* [23]. More recently, genome scans have narrowed the cis-regulatory region driving variation to a 65-kb region about 100-kb downstream of *optix* [31]. Phylogenetic analyses targeting this genomic region cluster *H. erato* races by color pattern, suggesting a single origin of the two major color pattern types within *H. erato* [31, 32].

Here, we leverage the recent progress in identifying the genomic region responsible for warning color variation to examine how genomes diverge through the speciation process. Specifically, we sample genomic variation across four geographically distinct hybrid zones between *H. erato* color pattern races and one hybrid zone between the proposed incipient species, *H. himera* and *H. e. cyrbia*. First, we reconstruct the evolutionary relationship of *H. himera* and races of *H. erato*. We use this phylogeny and a literature review of hybridization and reproductive barriers between *H. erato* races and *H. himera* to demonstrate the speciation continuum across the *H. erato* clade (Fig. 1). Then by sampling hybridizing races and species that reflect distinct points along this continuum, we empirically explore genomic divergence during the speciation process.

## Methods

### Sampling the *H. erato* speciation continuum

We examined phenotypically pure samples from five hybrid zones across the *H. erato* radiation with varying degrees of reproductive isolation. The first four hybrid zones are all between hybridizing races of *H. erato* that show no evidence of pre-mating or post-mating isolation [8, 10, 33]. One of these hybrid zones is between two different postman phenotypes (*H. e. hydara* x *H. e. petiverana* in Panama, *n* = 8). The other three are between divergent postman and rayed phenotypes (*H. e. erato* × *H. e. hydara* in French Guiana, *n* = 13; *H. e. emma* × *H. e. favorinus* in Peru, *n* = 14; and *H. e. lativitta* × *H. e. notabilis* in Ecuador, *n* = 10). For these four within-species hybrid zones, we utilized previously published data [GenBank:SRA059512] [31]. We have added to these data samples from a fifth hybrid zone between *H. himera* and *H. erato cyrbia*, two putative incipient species with pre-mating isolation, but no intrinsic post-mating isolation. We collected phenotypically pure samples of *H. himera* (*n* = 5) and *H. e. cyrbia* (*n* = 4) on either side of a narrow region of hybridization in Loja Province, Ecuador. We also collected samples from two outgroup species, *H. clysonymus* (*n* = 2) and *H. telesiphe* (*n* = 2) in Peru. Sampling locations are listed in Additional file 1: Table S1.

Within these intraspecific and interspecific hybrid zones, gene flow is known to occur based on the presence of hybrid individuals with wing color patterns that result from genetic backcrosses (e.g. individuals homozygous at one color pattern locus, but heterozygous at another) [8, 17]. However, each hybrid zone sampled is geographically isolated from one another, with some on opposite sides of South America and separated by major topographic features. This geographic isolation severely restricts gene flow between the races from different hybrid zones.

### Sequencing and genotyping

The samples from the *H. himera/H. e. cyrbia* hybrid zone and the outgroup taxa were sequenced and genotyped as described in [31]. Briefly, this involved whole genome sequencing of 100-bp paired end reads on the Illumina HiSeq platform and aligning reads to a partial genomic reference (2.2-Mb, ~0.5 % of the *H. erato* genome) [GenBank:KC469892-KC469895, AC208805-AC208806] with BWA v0.59-r16 [34] using relaxed mapping parameters. Hybrid zone samples were each sequenced to a realized median per base coverage between 13× and 21× (Additional file 1: Table S1). Outgroup species samples were sequenced to a realized median coverage of 8X or higher, due to a lower proportion of sequence reads mapping to the *H. erato* reference. Multi-sample genotypes were then called and filtered with GATK v1.2 [35, 36]. Aligned sequencing reads are available at NCBI SRA [GenBank:SRA059521] and genotype VCF files are available at Dryad [doi:10.5061/dryad.5n10d]. We examined within species genetic variation using four previously published parapatric *H. erato* racial hybrid zones [GenBank:SRA059512] [31]. Sequence variation for the newly sequenced and previously sequenced samples (*n* = 58 combined) was analyzed across a 1-Mb region containing the locus controlling red color pattern variation [GenBank:KC469894] and 350-kb from three additional genomic regions unlinked to color pattern loci [GenBank:KC469892, AC208805, AC208806].

### Phylogenetic analysis

We performed phylogenetic analyses of all the genomic data to (i) determine the phylogenetic relationship of *H. himera* and *H. erato*, which has previously been disputed [19–21], and (ii) use the phylogenetic relationships to develop expectations for genetic divergence between taxa. We generated phylogenetic consensus trees from 5,399 SNPs across the previously identified 65-kb region that controls red color pattern variation [31] and 15,714 SNPs across the 350-kb of genomic regions unlinked to color pattern. To reduce the size of the unlinked dataset, we removed invariant sites.

We selected nucleotide substitution models for both datasets using a hierarchical likelihood ratio test implemented in PAUP* v4 [37] and MrModeltest v2 [38]. We used MrBayes v3.2.2 [39] to generate consensus trees with the selected models—GTR + I + G for the 65-kb functional region and GTR + G for the unlinked loci. For each tree, we ran 5 runs for 5 million generations each, sampling every 500 generations and removing 25 % burn-in. We assessed burn-in and convergence in MrBayes and Tracer v1.6 [40]. For the unlinked tree, we removed two runs that converged to a slightly lower likelihood than the other three runs. We generated consensus trees from converged runs and rooted the trees with the *H. clysonymus* and *H. telesiphe* samples. Tree files are available at Dryad [doi:10.5061/dryad.5n10d%5D. We accounted for gene flow and phylogenetic conflict in inferring relationships by constructing an unrooted neighbor-net splits tree network on the unlinked dataset using SplitsTree v4.13.1 [41]. This analysis utilized pairwise distances and treated polymorphisms additively, rather than as ambiguities. We performed additional analyses to assess potential causes of the long branch lengths leading to the *H. himera* lineage. Specifically, we tested if the long branches leading to *H. himera* could be the result of different rates of evolution. We used BEAST v1.7.5 [42] to reconstruct the evolutionary relationships of *H. himera* and *H. erato* samples based on the 15,714 SNPs across the genomic regions unlinked to color pattern using i) a relaxed molecular clock and ii) a strict molecular clock. We used a GTR + I + G (four rate categories) model, estimated base frequencies, and a Yule tree prior. For each tree, we ran 2 runs for 10 million generations each, sampling every 5000 generations and removing 10 % burn-in. We compared the likelihood distributions from the relaxed clock and the strict clock using BayesFactors (1,000 bootstrap replicates) in Tracer version 1.5 and visually compared the relaxed clock and the strict clock consensus phylogenies, each constructed from 18,000 sampled trees.

### Genomic divergence analysis

We compared patterns of divergence between the incipient species, *H. himera* and *H. e. cyrbia* (between species comparison), to patterns of divergence among hybridizing races (within species, parapatric comparisons) and non-hybridizing races (within species, allopatric comparisons). The within species parapatric comparisons consisted of three *H. erato* hybrid zones between postman and rayed phenotypes (Peru, Ecuador, and French Guiana) and one *H. erato* hybrid zone between two different postman phenotypes (Panama). The within species allopatric comparisons consisted of all remaining pairwise comparisons between these same *H. erato* races, with the two Panamanian postman races being treated as a single race. These comparisons can be divided into three categories (postman *versus* rayed, *n* = 9 comparisons; postman *versus* postman, *n* = 6 comparisons; rayed *versus* rayed, *n* = 3 comparisons). Note that *H. e. cyrbia* is only included in comparison to *H. himera. Heliconius erato cyrbia* is not included in the within *H. erato* comparisons due to interspecific hybridization and the possibility of divergent *H. himera* haplotypes in *H. e. cyrbia* individuals inflating the within species divergence estimates.

For each comparison, we calculated relative divergence per genomic position using a model for diploid data with populations as random effects $\left(\widehat{\theta}\right)$, a commonly used estimate of *F*_*ST*_ [43], with implementation details described in [31]. In addition to relative divergence, for each comparison we also calculated absolute divergence (d_xy_) [44, equation 10.20]. For both analyses, we filtered data to remove positions where less than 75 % of individuals were genotyped for each of the two taxa being compared. To properly combine estimates across loci and across comparisons for relative divergence, the numerators and denominators are each summed across the pairwise estimates and then divided to produce the final combined estimate. To combine estimates for absolute divergence, the mean was used. We calculated the genomic background level of relative divergence from three genomic intervals (0.35-Mb) unlinked to color pattern, using 1000 bootstrap replicates to determine 95 % confidence intervals (CIs). For sliding window analyses, we examined 15-kb windows, with 5-kb steps, requiring estimates for at least 20 % of the positions in the window.

To examine the decay of divergence with recombination distance, we defined the causative locus as the center of the 65-kb region previously identified to modulate red color pattern variation in *H. erato* [31]. We converted genomic distance (bp) to recombination distance (cM) assuming a constant recombination rate based on the *H. erato* linkage map size (1430 cM) [45] and the estimated size of the *H. erato* genome (400 Mb) [46], which provides an average estimate of recombination rate across the genome. Loci were binned by recombination distance from the causative locus in bins of size 0.01 cM. Decay data were loess smoothed for presentation.

## Results and Discussion

### *Heliconius himera*—an incipient species from within the *H. erato* radiation

**Fig. 2:**
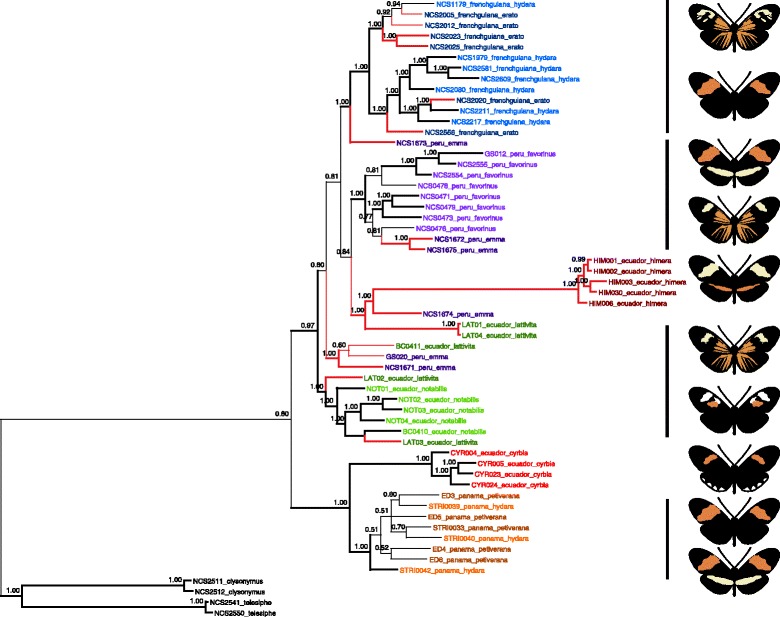
Bayesian phylogenetic relationships at loci unlinked to color pattern. Phylogenetic clustering sorts taxa strongly by geographic location. There is a strong phylogenetic break across the Andes, with *H. himera* clustering with *H. erato* races from the eastern slopes of the Andes, while *H. e. cyrbia* clusters with races from the western slopes of the Andes. *Heliconius himera* clusters within *H. erato*, rather than diverging prior to the *H. erato* radiation. Red branches indicate rayed lineages. Nodal values are posterior probabilities of clade support, with bold braches indicating a node with >95 % support. Sample names (ID, hybrid zone, race) are indicated at the terminal nodes, color-coded by race

**Fig. 3:**
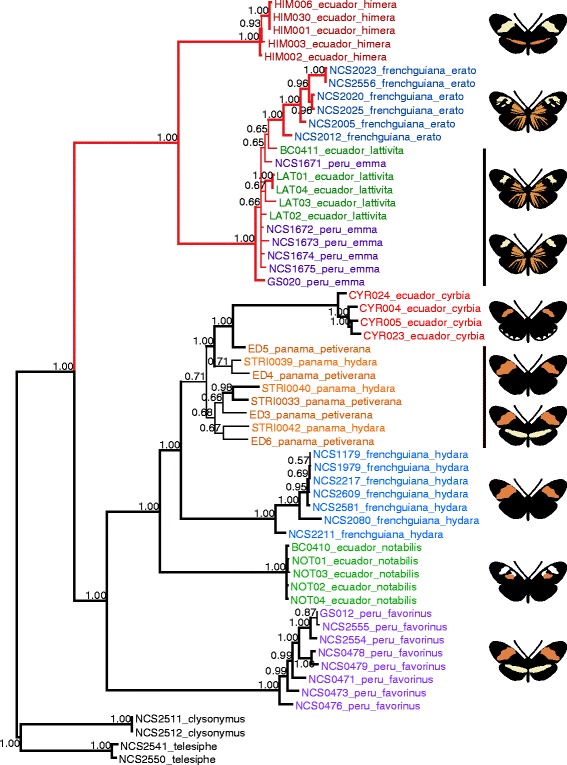
Bayesian phylogenetic relationships at the red color locus. Phylogenetic clustering across the 65-kb region that modulates red color pattern variation sorts taxa by phenotype. The postman and the rayed clades form reciprocally monophyletic lineages, with *H. himera* clustering with the rayed races, while *H. e. cyrbia* clusters with the postman races. Within each phenotype, samples cluster by geography. *Heliconius himera* clusters within *H. erato*, rather than diverging prior to the *H. erato* radiation. Red branches indicate rayed lineages. Nodal values are posterior probabilities of clade support, with bold braches indicating a node with >95 % support. Sample names (ID, hybrid zone, race) are indicated at the terminal nodes, color-coded by race

Our phylogenetic analysis clearly places *H. himera* within the *H. erato* radiation, both at the color pattern locus and at loci unlinked to color pattern (Figs. 2 and 3). These results indicate that *H. himera* evolved from within the broader *H. erato* color pattern radiation, rather than predating it. The Bayesian phylogenetic tree of *H. erato* races across loci unlinked to color pattern shows a strong geographic signal, with no clustering by color pattern (Fig. 2). Similar to previous research, there is a strong phylogenetic break across the Andes, with taxa on the eastern slopes of the Andes clustering separately from taxa on the western slopes [32, 47]. All five *H. himera* individuals fall on a well-differentiated lineage that clusters with *H. erato* races from the eastern slopes of the Andes and thus within the *H. erato* radiation (Fig. 2).

Given the long branch length and weaker support values leading to the *H. himera* lineage, we used additional methods to assess the support for *H. himera* evolving from within the *H. erato* radiation *versus* predating it. We reran the Bayesian analysis with invariant sites retained, but filtered out sites with too much missing data. The tree topology did not change, nor did the long branch lengths leading to the himera lineage (results not shown). The splits tree shows *H. himera* as a strongly monophyletic lineage (i.e. with a long branch length preceding it) and nested within the *H. erato* radiation, with closer affinity for the *H. erato* taxa from the eastern slopes of the Andes than the taxa from the western slopes (Additional file 2: Figure S1). These network plots suggest there may be some phylogenetic conflict arising from allele sharing between *H. himera* and *H. e. cyrbia*, as evidenced by some of the alternative connections at the base of the topology. However, the combined evidence strongly allies *H. e. cyrbia* to the western lineage, distinctly separate from *H. himera*, with a low signal of genetic admixture between the two. Collectively, our analyses consistently support *H. himera* as part of the *H. erato* radiation, with a close association to *H. erato* samples from east of the Andes. This placement of *H. himera* was further supported by the phylogenetic reconstructions using a relaxed molecular clock in BEAST. The relaxed molecular clock showed a much better fit to the data than a strict clock (Additional file 3: Figure S2). This suggests that the long branches may be due to a more rapid accumulation of substitutions in the *H. himera* lineage relative to *H. erato.*

A very different phylogenetic history was observed across the genomic region that controls red color pattern variation, reflecting the evolution of the color pattern alleles. This tree strongly clusters taxa by color pattern, showing two reciprocally monophyletic clades sorting perfectly by red phenotype (Fig. 3). The “postman” clade contains all of the *H. erato* postman races, including *H. erato cyrbia*, while the “rayed” clade contains all of the *H. erato* rayed races and *H. himera*. Even though *H. himera* does not have the characteristic rays, it is phenotypically more similar to the rayed phenotypes since they share a number of wing color pattern elements, including the presence of red on the hindwing. This classification of *H. himera* as being most similar to the *H. erato* rayed is supported by our phylogenetic analysis.

While the analyses at loci unlinked to color pattern and across the genomic region containing the functional variants show different patterns of clustering (by geography and phenotype, respectively), both place *H. himera* within the *H. erato* radiation, supporting *H. himera* as an incipient species derived from within *H. erato*. For the incipient species pair, *H. himera* and *H. e. cyrbia*, each fall into different monophyletic lineages at both color pattern-linked and unlinked loci. For unlinked loci, *H. e. cyrbia* is in the western clade and *H. himera* is in the eastern clade, and for the color locus, *H. e. cyrbia* falls with the postman races and *H. himera* with the rayed races. From an experimental perspective, the presence of two well-supported monophyletic lineages for each of these analyses suggests that taxa pairs sampled from each of these lineages are expected to have comparable divergence times to the incipient species pair. This enables us to make comparisons between levels of genomic divergence for the incipient species pair and other taxa pairs from the two lineages without having to control for divergence times.

### Selection drives divergence at color pattern loci

**Fig. 4:**
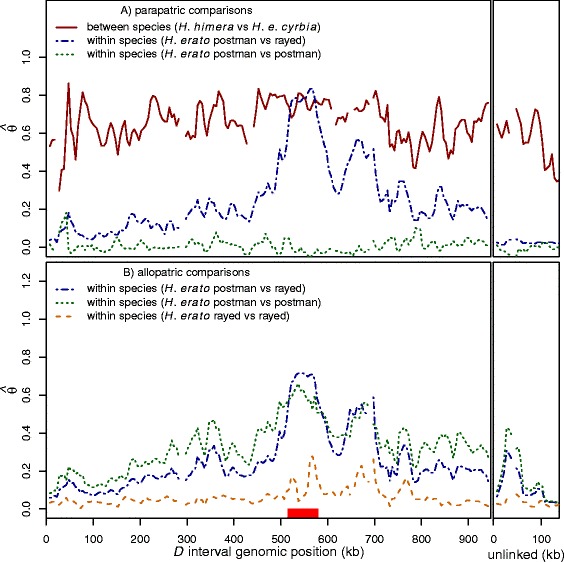
Relative genomic divergence across the red color pattern (*D*) interval and unlinked loci. Sliding window (15-kb window size, 5-kb step size) relative genomic divergence across the red (*D*) interval and genomic regions unlinked to color pattern. The box on the x-axis indicates the previously identified 65-kb functional region modulating red color pattern variation [31]. See Table S2 for the taxa pairs included in each comparison, samples sizes, and estimates of baseline divergence from intervals unlinked to color pattern. **a** Parapatric comparisons show elevated divergence between the incipient species (solid line) at the color pattern locus and loci unlinked to color pattern. The within species comparisons between divergent taxa show a large peak of divergence at the functional region and low baseline divergence (dotted-dashed line). The within species comparisons between taxa with identical red phenotypes show low divergence throughout, with no discernable peaks of divergence (dotted line). **b** The allopatric within species comparisons show peaks of divergence at the functional region between both divergent taxa and mimetic taxa, with the rayed *versus* rayed comparison (dashed line) showing much smaller peaks

Our analysis of relative divergence shows distinct peaks at the functional regions that modulate adaptive differences in wing color patterns between hybridizing *H. erato* races (Fig. 4a). As previously demonstrated, when comparing between parapatric postman and rayed races, there are two distinct elevated peaks of divergence surrounded by steep drop-offs almost all the way to the near zero genomic baseline divergence [31]. These peaks are in sharp contrast to (i) the near zero divergence observed across the red color pattern locus between parapatric races that share similar red color patterns (parapatric postman *versus* postman comparison, Fig. 4a) and (ii) the broad elevated relative divergence between the incipient species that has no clear peak (parapatric between species comparison, Fig. 4a). We believe that these peaks of divergence are being driven by strong selection on wing color pattern. The similarity of the peaks between the *H. erato* hybrid zones demonstrates the ability of selection to drive similar patterns of divergence among different populations, when the populations are under similar selective pressures.

All of the comparisons between non-hybridizing allopatric races show peaks of relative divergence around the locus modulating red color pattern variation, including comparisons between races that share the same red phenotype (Fig. 4b). The pattern of divergence among allopatric postman *versus* postman is particularly noteworthy, as it shows strong divergence peaks similar to those observed between hybridizing races with different red color patterns. Similar peaks of relative divergence are also found in the allopatric rayed *versus* rayed comparisons, albeit the peaks are small. These peaks of relative divergence between similar colored, allopatric races could reflect the history of strong selection at each hybrid zone against the introgression of divergent color patterns. This strong selection on color pattern will also affect allele frequencies at neutral loci that are tightly linked to sites that modulate the color pattern variation. We propose that selection at each hybrid zone may have lead to the fixation of different neutral alleles near the causative sites of the red locus, at each hybrid zone.

Comparisons between patterns of absolute and relative divergence can be used to better understand the role that gene flow and selection play in generating patterns of genomic divergence [48]. Peaks of relative divergence may not always result from divergent selection between hybridizing groups. High relative divergence between two groups can also be the result of reduced diversity due to selection within the populations, even in the absence of gene flow [49, 50]. Overall we found strikingly similar patterns of relative and absolute divergence in each of the hybrid zones. However, absolute divergence was noisier than relative divergence in all comparisons (Additional file 4: Figures S3 and Fig. 4). For all hybridizing taxa that differ in red pattern, there was a clear peak of absolute and relative divergence near the red locus (Additional file 4: Figure S3A). This was expected since we know that all the hybridizing taxa experience ongoing gene flow and strong divergent selection on red wing color patterns. Between the non-hybridizing taxa, the patterns of relative and absolute divergence were not as consistent. Between the allopatric postman races, there were clear peaks of relative and absolute divergence at the color pattern locus (Additional file 4: Figure S3C). These peaks between similarly colored allopatric races likely reflect the history of selection and genetic hitchhiking at each hybrid zone. For example, selection against the introgression of the postman haplotype at the red locus from the postman race into the rayed race in a hybrid zone could result in genetic hitchhiking of neutral alleles at several sites near the red locus. Similarly, selection against the introgression of the postman haplotype at the red locus from the postman race into the rayed race in a different hybrid zone could result in genetic hitchhiking of neutral alleles at several sites near the red locus that could be different from the sites that experienced genetic hitchhiking in the first hybrid zone. This could result in a relative excess of allelic differences at several sites near the red locus between the similarly colored races at the two hybrid zones, as we observed in the allopatric postman comparisons. However, this cannot explain the allopatric rayed race comparisons where we found a reduction of absolute divergence near the red locus (Additional file 4: Figure S3D), and only a slight peak of relative divergence (Fig. 4b). These patterns may reflect the relatively recent origin of the rayed phenotype [32] and/or the greater potential for gene flow among the rayed races due to their contiguous distribution across the Amazon basin [51].

Collectively, these results add to a growing body of evidence that demonstrate that selection *within* populations can leave the same signature of selection as ecological divergence *between* populations [48, 52, 53]. Given the complex patterns of divergence resulting from the interactions between various evolutionary forces, peaks of divergence should be interpreted with caution, especially when additional supporting data are not available.

### Divergence accumulates rapidly during speciation

The phylogenetic placement of *H. himera* within the broader *H. erato* radiation allows us to compare patterns of genomic divergence at different points along the speciation continuum. Genomic divergence between the incipient species *H. himera* and *H. e. cyrbia* is substantially higher than the divergence seen in all other comparisons. The divergence is much higher across the 1-MB interval, except at the narrow region containing the functional variation where most comparisons show very high divergence (Fig. 4). The unlinked loci also show high divergence between the incipient species (average = 0.519, 95 % CI = 0.507-0.531), significantly higher than any of the within species comparisons (maximum average = 0.191, 95 % CI = 0.182-0.200) (Additional file 1: Table S2). This is a relatively high level of divergence given that *H. himera* is a recent incipient species and is known to hybridize frequently with *H. erato* in nature (Figs. 2 and 3, [16–18]). These patterns suggest that divergence may accumulate rapidly in the early stages of speciation, even if reproductive isolation is incomplete and gene flow is still occurring. Some previous work in other *Heliconius* clades has also shown that genomic divergence can occur rapidly as speciation progresses [54, 55]; however, one study of other hybridizing *Heliconius* species suggested divergence may build gradually over long periods of continuous gene flow [56]. Our results are more consistent with the former *Heliconius* studies, as well as empirical studies in other systems that report high genome wide divergence early in speciation, even in the face of ongoing gene flow [57, 58].

### What drives high divergence between incipient species?

Our experimental design allows us to begin to disentangle the effects of selection and gene flow on patterns of genomic divergence during speciation. By comparing levels of divergence between hybridizing and non-hybridizing races, we can empirically see the effects of differing levels of gene flow. As expected, non-hybridizing races (allopatric) show higher background levels of genomic divergence than hybridizing races (parapatric) (Additional file 5: Figure S4, blue shaded area [0.042-0.173] *versus* green shaded area [0.020-0.031]). Comparisons of non-hybridizing races provide an estimate of the expected level of divergence between divergently colored taxa with limited gene flow. Since, the phylogenetic reconstructions showed that *H. himera* and *H. cyrbia* belong to different clades at the color locus and color unlinked loci, we may expect similar levels of genomic divergence to have accumulated between the incipient species, as have accumulated between non-hybridizing races from the two different clades.

In contrast to this expectation, genomic divergence between the incipient species is much higher than between the non-hybridizing races (Fig. 4 and Additional file 5: Figure S4). Divergence between the incipient species was not only higher across the color pattern locus, but also across the genomic regions unlinked to color pattern variation. For instance, the background divergence between non-hybridizing races with different color patterns from Peru and French Guiana (0.191; 95 % CI: 0.182-0.200) is significantly lower than the divergence between the incipient species (0.519; 95 % CI: 0.507-0.531), despite higher realized rates of gene flow between the incipient species than the non-hybridizing races. *H. himera* and *H. e. cyrbia* hybridize continuously and multi-generational hybrids (based on color pattern genotypes) can readily be found where they overlap geographically. This suggests that there is likely genomic admixture occurring every generation between the incipient species. In contrast, the non-hybridizing races sampled are often thousands of kilometers apart (e.g. Peru and French Guiana are located on opposite sides of South America) and never come into direct contact. Even though *H. erato* does have a wide, continuous distribution across South America, the lifetime dispersal distance for *H. erato* is estimated to be only ~5 km per generation [59], which suggests admixture between races thousands of kilometers apart would take hundreds of generations to occur. Therefore, despite the expectation that higher gene flow should homogenize genetic variation and decrease divergence across the genome, we find that divergence is higher between the taxa that also have greater potential for gene flow.

The higher levels of divergence between *H. himera* and *H. e. cyrbia* likely reflect unique selective pressures and/or population histories. These differences could simply be the result of population bottlenecks and/or periods of allopatry that allowed divergence to rapidly accumulate between the populations, and the hybridization is due to recent secondary contact. Alternatively, these patterns could be the result of natural selection rapidly driving genomic divergence, even with continuous gene flow. Selection has been shown to play a primary role in speciation in both theoretical models [60] and empirical studies [see references in 1]. In particular, selection acting on many loci across the genome is suggested to result in an increase in genome wide divergence [61, 62]. Unlike geographic races of *H. erato*, *H. himera* and *H. e. cyrbia* show strong divergence beyond just color pattern, including differences in mating preference, larval developmental time, adult physiology, and habitat preferences [14–18, 63, 64]. Our results support the hypothesis that divergent selection on multiple traits and loci may drive the high divergence found between the incipient species *H. himera* and *H. e. cyrbia*. Our within species comparisons show that the effect of selection on color pattern loci is restricted to a small region of the genome, with the decay of relative divergence occurring between 1-2 cM from the causative locus (Additional file 5: Figure S4). This rapid rate of decay is what is predicted from models of divergence hitchhiking around a gene under strong selection [4]. Our results are consistent with several theoretical and empirical studies that report high genome wide divergence and a limited role for divergence hitchhiking in promoting speciation [54, 57, 58, 61, 62, 65, 66]. These results contrast with a study in the *Heliconius melpomene* clade, that suggested that divergence hitchhiking may play a prominent role during the early stages of speciation [56]. However, *H. erato* and *H. himera* show stronger evidence of habitat based ecological divergence than the incipient species studied in the *H. melpomene* clade [33]. Our results highlight a limited role of divergence hitchhiking and suggest that the increase in divergence seen between the incipient species likely requires genomic hitchhiking through selection at multiple loci.

## Conclusions

*Heliconius* butterflies provide a full continuum of taxa pairs at varying stages of reproductive isolation—from freely hybridizing color pattern races to completely reproductively isolated species. By sampling across this continuum, we can observe how genomes diverge through the speciation process and begin to disentangle the evolutionary forces that drive species divergence. Here, we resolve the phylogenetic placement of *H. himera* within the *H. erato* clade and demonstrate that *H. himera* shares a similar haplotype at the red color pattern locus to rayed *H. erato* races. This phylogenetic reconstruction demonstrates that the hybridizing color pattern races and incipient species sampled in this study reflect distinct points of divergence along the speciation continuum. Comparisons of population divergence at these different points in the continuum show that as reproductive isolation increases, genomic divergence increases, not only near the regions responsible for adaptive divergence and reproductive isolation, but also at regions unlinked to known adaptive loci. This occurs much earlier in the speciation process than previously envisioned. By examining different regions of the genome, we were able to provide a series of snapshots of divergence through the speciation process that suggest complex population histories and selective pressures likely drive rapid divergence early in the speciation process. The results also suggest that reference-genome-enabled, genome-wide population demographic analyses across the *H. erato* speciation continuum may provide valuable insights into the effects of selection and gene flow on the origin of species.

## Availability of supporting data

The data supporting the results of this article, including SNP calls and phylogenetic trees, are available in the Dryad repository [doi:10.5061/dryad.5n10d, http://datadryad.com]. Aligned sequencing reads are available at the NCBI Short Read Archive [GenBank:SRA059521 (SRX1176360, SRX1176362-3, SRX1176365-74), http://www.ncbi.nlm.nih.gov/sra]. Custom analysis scripts are available on GitHub [https://github.com/LaMariposa].

## Additional files

Additional file 1: Table S1. Samples and sequencing data. **Table S2.** Taxa pairs for divergence analysis. (PDF 140 kb) Additional file 2: Figure S1. Phylogenetic network at loci unlinked to color pattern. Neighbor-net splits tree network at loci unlinked to color pattern. Terminal nodes indicate voucher number, hybrid zone, and race of each sample. *Heliconius himera* shows a stronger affinity to taxa east of the Andes than the taxa from west of the Andes, which cluster separately. (PDF 62 kb) Additional file 3: Figure S2. Phylogenetic relationships under a relaxed clock. The phylogenetic tree generated when substitution rates among lineages are allowed to vary. The tree shows *H. himera* nested within the *H. erato* radiation. Results for Bayesfactor comparison of strict and relaxed molecular clock topologies are shown in upper left corner. (PNG 109 kb) Additional file 4: Figure S3. Absolute genomic divergence across the red color pattern (*D*) interval and unlinked loci. Sliding window (15-kb window size, 5-kb step size) absolute genomic divergence (d_xy_) across the red (*D*) interval and genomic regions unlinked to color pattern. See Additional file 1: Table S2 for the taxa pairs included in each comparison and samples sizes. (A) Parapatric hybrid zones. (B) Allopatric postman *versus* rayed comparisons. (C) Allopatric postman *versus* postman comparisons. (D) Allopatric rayed *versus* rayed comparisons. (PDF 29 kb) Additional file 5: Figure S4. Decay of divergence with recombination distance. The curves represent the decay of genomic divergence with distance from the causative color pattern locus between taxa pairs with divergent phenotypes. We converted genomic distance (bp) to recombination distance (cM) assuming a constant recombination rate based the *H. erato* linkage map size (1430 cM) [45] and the estimated size of the *H. erato* genome (400 Mb) [46], thus assuming a constant recombination rate across the genomic regions and across taxa. The extent of variation in recombination rate in *Heliconius* is unknown, as such this estimate merely provides a rough estimate of recombination distance from the causative locus. See Additional file 1: Table S2 for the taxa pairs included in each comparison, samples sizes, and estimates of baseline divergence from intervals unlinked to color pattern. The results were loess smoothed for presentation, which potentially introduces additional biases in the estimates of rate of decay. The comparisons are between incipient species (*H. himera versus H. e. cyrbia*; red dashed), average of within species parapatric postman *versus* rayed pairs (green dotted-dashed), and average of within species allopatric postman *versus* rayed pairs (blue dotted). The horizontal red dashed line represents the background genomic divergence between the incipient species at loci unlinked to color pattern. The blue and green shaded boxes are the range of baseline divergences at unlinked loci for within species allopatric and parapatric comparisons, respectively. The divergence at both the color pattern locus and unlinked genomic regions is substantially higher between species than it is within species. (PDF 5 kb)
