# Supplementary material for: Divergence with gene flow across a speciation continuum of Heliconius butterflies
Source: BMC Evol Biol. 2015 Sep 24;15:204. doi: 10.1186/s12862-015-0486-y (PMC4582928; doi:10.1186/s12862-015-0486-y)
Supplement: Additional file 1: Table S1. — Samples and sequencing data. Table S2. Taxa pairs for divergence analysis. (PDF 140 kb) [file 12862_2015_486_MOESM1_ESM.pdf]

Supplemental Tables (Supple et al. 2015, Divergence with gene flow across a speciation continuum of *Heliconius* butterflies)
Table S1: Samples and sequencing data

taxa (location)	sample ID	geolocation	number of paired end reads	mapped reads (%)	properly mapped pairs (% of mapped)	median coverage	positions genotyped (%)		SNPs* per genotyped position (%)	
							all reference	*D* interval	all reference	*D* interval
H. himera (Ecuador)	HIM001	04°16'34''S 79°11'45''W	47953654	8.8	71.7	17	45.8	51.7	4.3	3.6
	HIM002	04°16'34''S 79°11'45''W	52114768	8.8	71.4	19	47.8	54.2	4.4	3.7
	HIM003	04°16'34''S 79°11'45''W	54102534	8.5	71.7	20	49.3	55.5	4.9	3.8
	HIM006	04°16'34''S 79°11'45''W	38797900	8.8	70.8	14	41.8	47.5	4.1	3.5
	HIM030	04°16'34''S 79°11'45''W	36866545	8.8	70.2	13	39.7	45.3	4.1	3.3
H. erato cyrbia (Ecuador)	CYR004	03°43'35''S 79°50'12''W	58110947	10.8	74.2	21	52.0	59.4	4.8	3.7
	CYR005	03°43'35''S 79°50'12''W	53098890	10.7	74.0	20	50.2	57.3	4.5	3.5
	CYR023	03°43'35''S 79°50'12''W	52694366	9.9	72.1	20	52.4	59.2	4.7	3.6
	CYR024	03°43'35''S 79°50'12''W	52080755	10.9	74.3	19	49.3	56.6	4.6	3.5
H. clysonymus (Peru)	NCS2511	00°42'46''S 77°44'27''W	45312108	7.1	63.5	8	25.0	28.4	5.0	4.1
	NCS2512	00°42'46''S 77°44'27''W	54844792	7.6	65.3	9	28.1	32.2	5.5	4.6
H. telesiphe (Peru)	NCS2541	00°43'04''S 77°40'56''W	53997408	7.3	66.4	10	28.9	33.6	5.3	4.4
	NCS2550	00°42'46''S 77°44'27''W	59716322	7.1	65.0	12	32.2	37.0	5.7	4.8

*SNPs are variation relative to the reference genome

Table S2: Taxa pairs for divergence analyses

geographic relationship	phenotypic comparison	taxon 1 name (location)	taxon 1 phenotype	taxon 1 sample size	taxon 2 name (location)	taxon 2 phenotype	taxon 2 sample size	baseline differentiation (95% CI)
parapatric	himera vs cyrbia	*H. himera* (Ecuador)	rayed	5	*H. e. cyrbia* (Ecuador)	postman	4	0.519 (0.507, 0.531)
	postman vs rayed	*H. e. emma* (Peru)	rayed	6	*H. e. favorinus* (Peru)	postman	8	0.022 (0.019, 0.025)
		H. e. lativitta (Ecuador)	rayed	5	*H. e. notabilis* (Ecuador)	postman	5	0.031 (0.028, 0.035)
		H. e. erato (French Guiana)	rayed	6	*H. e. hydara* (French Guiana)	postman	7	0.020 (0.017,0.023)
	postman vs postman	*H. e. petiverana* (Panama)	postman	5	*H. e. hydara* (Panama)	postman	3	0.001 (0.000, 0.006)
allopatric	postman vs postman	*H. e. favorinus* (Peru)	postman	8	*H. e. notabilis* (Ecuador)	postman	5	0.061 (0.057, 0.066)
		H. e. favorinus (Peru)	postman	8	*H. e. hydara* (French Guiana)	postman	7	0.079 (0.074, 0.084)
		H. e. favorinus (Peru)	postman	8	*H. e. petiverana* & *H. e. hydara* (Panama)	postman	8	0.191 (0.182, 0.200)
		H. e. notabilis (Ecuador)	postman	5	*H. e. hydara* (French Guiana)	postman	7	0.092 (0.086, 0.098)
		H. e. notabilis (Ecuador)	postman	5	*H. e. petiverana* & *H. e. hydara* (Panama)	postman	8	0.161 (0.152, 0.169)
		H. e. hydara (French Guiana)	postman	7	*H. e. petiverana* & *H. e. hydara* (Panama)	postman	8	0.174 (0.165, 0.182)
	rayed vs rayed	*H. e. emma* (Peru)	rayed	6	*H. e. lativitta* (Ecuador)	rayed	5	0.027 (0.024, 0.031)
		H. e. emma (Peru)	rayed	6	*H. e. erato* (French Guiana)	rayed	6	0.048 (0.044, 0.052)
		H. e. lativitta (Ecuador)	rayed	5	*H. e. erato* (French Guiana)	rayed	6	0.053 (0.049, 0.058)

Table S2 (continued)

geographic relationship	phenotypic comparison	taxon 1			taxon 2			baseline differentiation (95% CI)
		name (location)	phenotype	sample size	name (location)	phenotype	sample size	
allopatric	postman vs rayed	*H. e. emma* (Peru)	rayed	6	*H. e. notabilis* (Ecuador)	postman	5	0.047 (0.042, 0.051)
		H. e. emma (Peru)	rayed	6	*H. e. hydara* (French Guiana)	postman	7	0.072 (0.067, 0.077)
		H. e. emma (Peru)	rayed	6	*H. e. petiverana* & *H. e. hydara* (Panama)	postman	8	0.161 (0.152, 0.169)
		H. e. lativitta (Ecuador)	rayed	5	*H. e. favorinus* (Peru)	postman	8	0.052 (0.048, 0.056)
		H. e. lativitta (Ecuador)	rayed	5	*H. e. hydara* (French Guiana)	postman	7	0.079 (0.075, 0.085)
		H. e. lativitta (Ecuador)	rayed	5	*H. e. petiverana* & *H. e. hydara* (Panama)	postman	8	0.159 (0.150, 0.168)
		H. e. erato (French Guiana)	rayed	6	*H. e. favorinus* (Peru)	postman	8	0.056 (0.052, 0.060)
		H. e. erato (French Guiana)	rayed	6	*H. e. notabilis* (Ecuador)	postman	5	0.068 (0.063, 0.073)
		H. e. erato (French Guiana)	rayed	6	*H. e. petiverana* & *H. e. hydara* (Panama)	postman	8	0.164 (0.156, 0.173)
