# Supplementary figures and images for: Divergence with gene flow across a speciation continuum of Heliconius butterflies
Source: BMC Evol Biol. 2015 Sep 24;15:204. doi: 10.1186/s12862-015-0486-y (PMC4582928; doi:10.1186/s12862-015-0486-y)

eastern clade

0.01

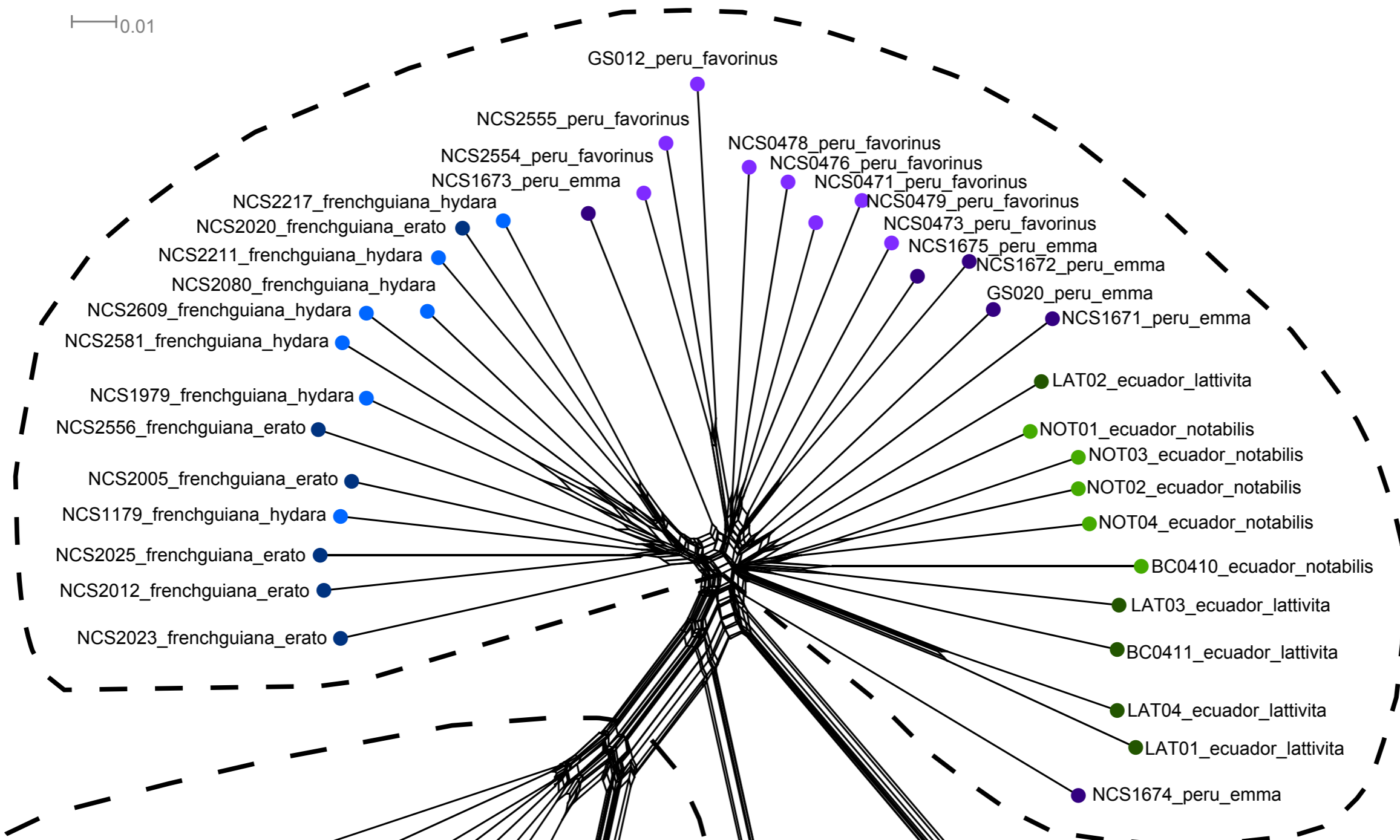

western clade

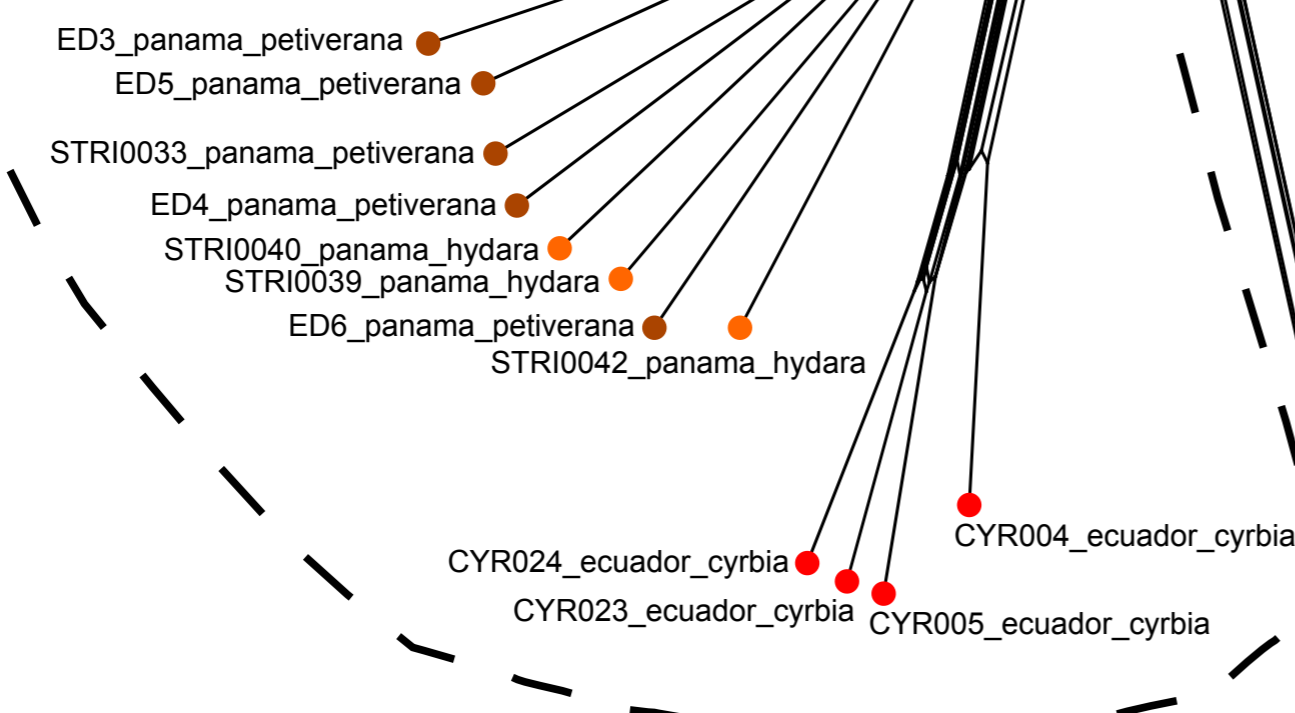

*H. himera*

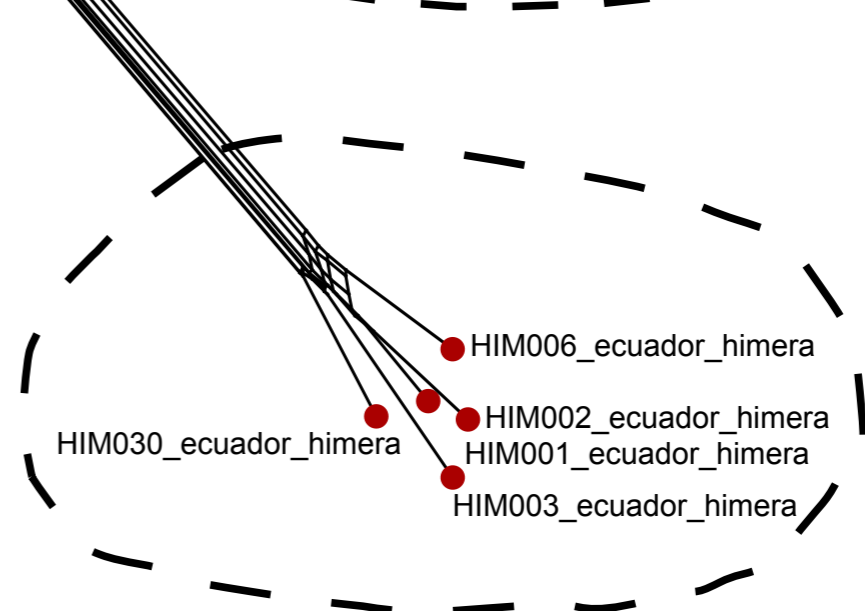

NCS2511\_clysonymus

NCS2512\_clysonymus

NCS2541\_tesiphe

NCS2550\_tesiphe

Supplement: Additional file 2: Figure S1. — Phylogenetic network at loci unlinked to color pattern. Neighbor-net splits tree network at loci unlinked to color pattern. Terminal nodes indicate voucher number, hybrid zone, and race of each sample. Heliconius himera shows a stronger affinity to taxa east of the Andes than the taxa from west of the Andes, which cluster separately. (PDF 62 kb) [file 12862_2015_486_MOESM2_ESM.pdf]

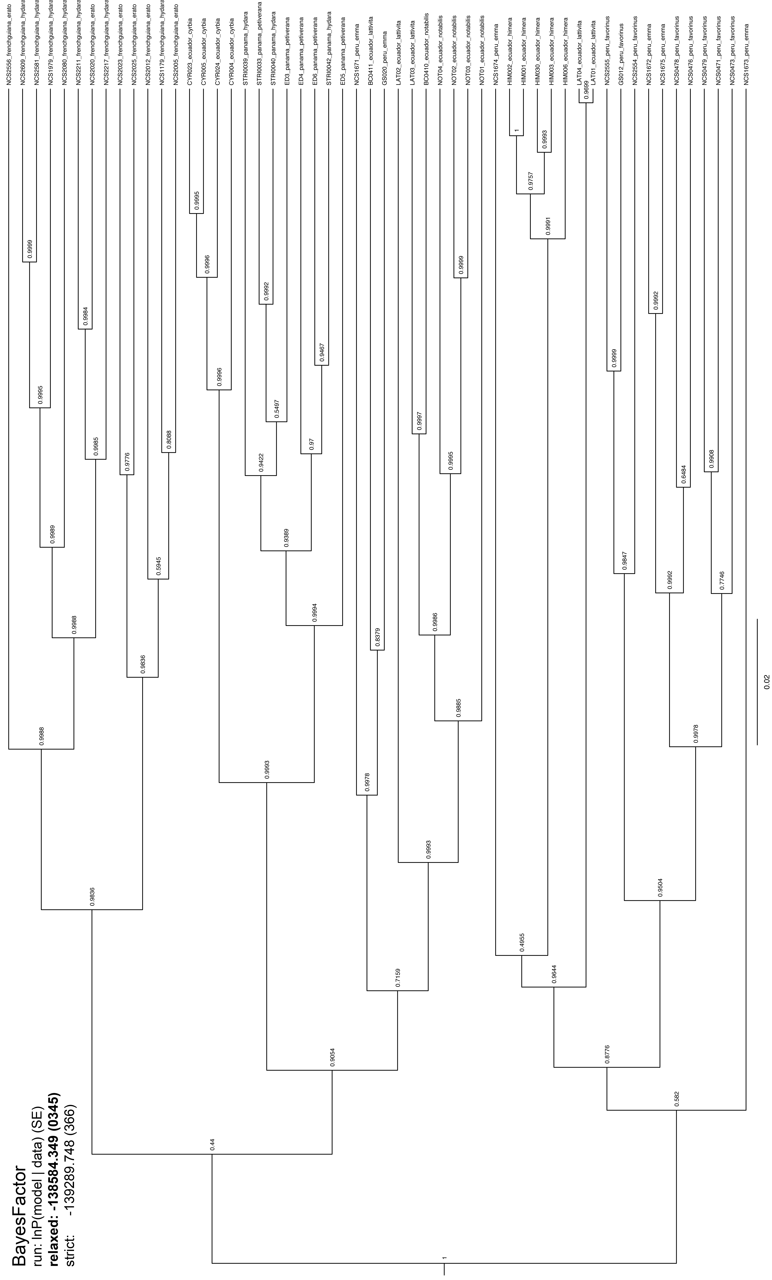

Supplement: Additional file 3: Figure S2. — Phylogenetic relationships under a relaxed clock. The phylogenetic tree generated when substitution rates among lineages are allowed to vary. The tree shows H. himera nested within the H. erato radiation. Results for Bayesfactor comparison of strict and relaxed molecular clock topologies are shown in upper left corner. (PNG 109 kb) [file 12862_2015_486_MOESM3_ESM.png]

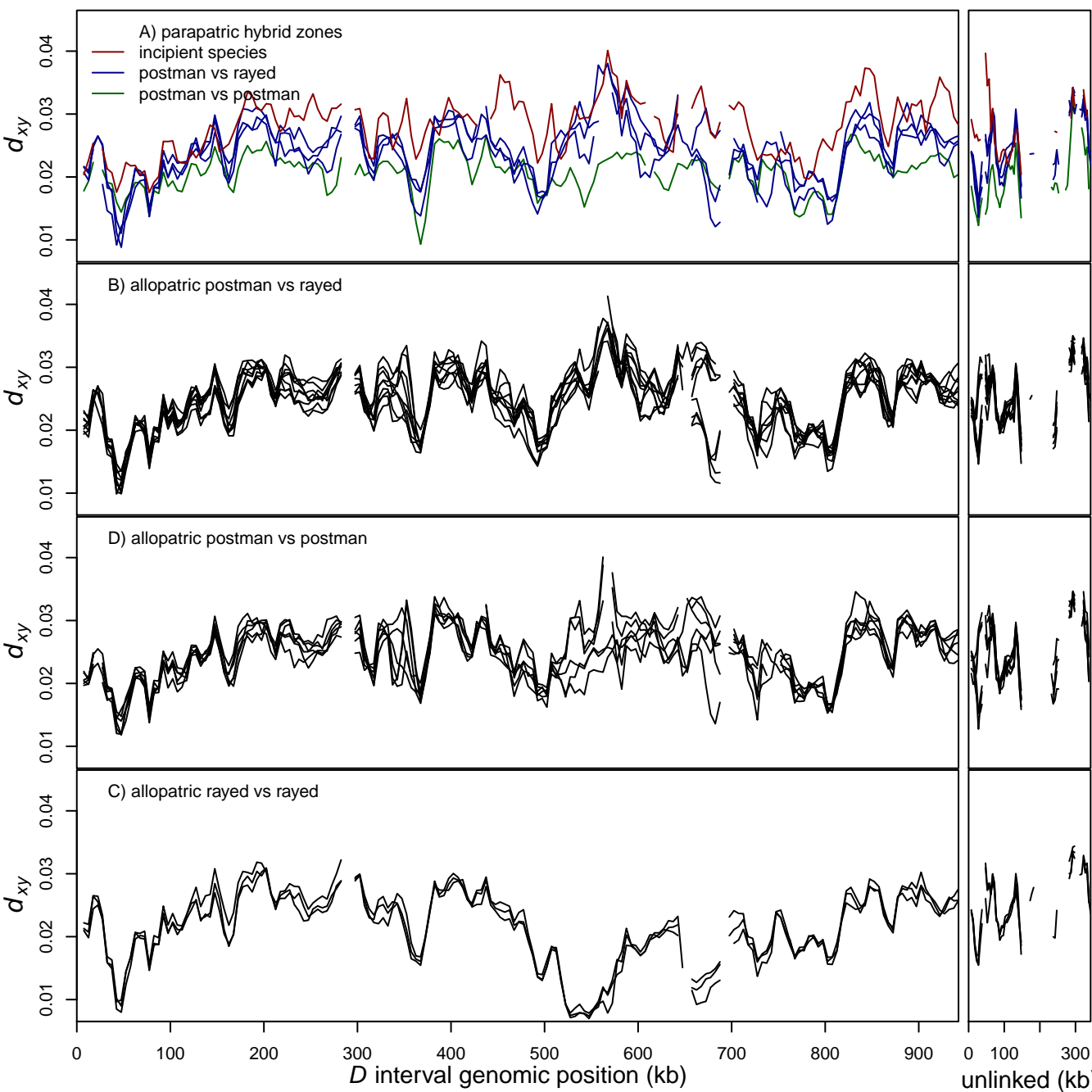

Supplement: Additional file 4: Figure S3. — Absolute genomic divergence across the red color pattern (D) interval and unlinked loci. Sliding window (15-kb window size, 5-kb step size) absolute genomic divergence (dxy) across the red (D) interval and genomic regions unlinked to color pattern. See Additional file 1: Table S2 for the taxa pairs included in each comparison and samples sizes. (A) Parapatric hybrid zones. (B) Allopatric postman versus rayed comparisons. (C) Allopatric postman versus postman comparisons. (D) Allopatric rayed versus rayed comparisons. (PDF 29 kb) [file 12862_2015_486_MOESM4_ESM.pdf]

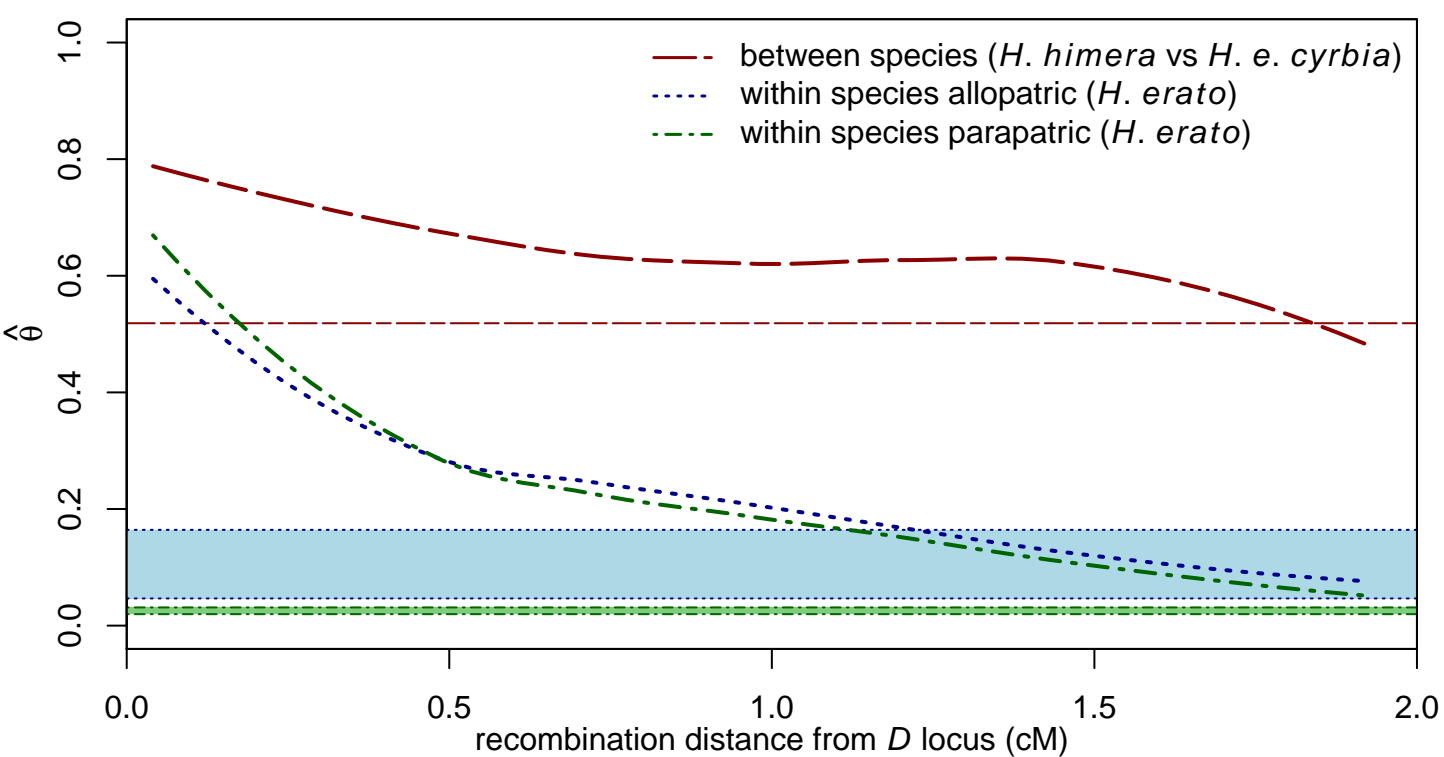

Supplement: Additional file 5: Figure S4. — Decay of divergence with recombination distance. The curves represent the decay of genomic divergence with distance from the causative color pattern locus between taxa pairs with divergent phenotypes. We converted genomic distance (bp) to recombination distance (cM) assuming a constant recombination rate based the H. erato linkage map size (1430 cM) [45] and the estimated size of the H. erato genome (400 Mb) [46], thus assuming a constant recombination rate across the genomic regions and across taxa. The extent of variation in recombination rate in Heliconius is unknown, as such this estimate merely provides a rough estimate of recombination distance from the causative locus. See Additional file 1: Table S2 for the taxa pairs included in each comparison, samples sizes, and estimates of baseline divergence from intervals unlinked to color pattern. The results were loess smoothed for presentation, which potentially introduces additional biases in the estimates of rate of decay. The comparisons are between incipient species (H. himera versus H. e. cyrbia; red dashed), average of within species parapatric postman versus rayed pairs (green dotted-dashed), and average of within species allopatric postman versus rayed pairs (blue dotted). The horizontal red dashed line represents the background genomic divergence between the incipient species at loci unlinked to color pattern. The blue and green shaded boxes are the range of baseline divergences at unlinked loci for within species allopatric and parapatric comparisons, respectively. The divergence at both the color pattern locus and unlinked genomic regions is substantially higher between species than it is within species. (PDF 5 kb) [file 12862_2015_486_MOESM5_ESM.pdf]
